# Increased [^18^F]DPA-714 Uptake in the Skeletal Muscle of SOD1^G93A^ Mice: A New Potential of Translocator Protein 18 kDa Imaging in Amyotrophic Lateral Sclerosis

**DOI:** 10.3390/biom15060799

**Published:** 2025-05-31

**Authors:** Cecilia Marini, Mattia Riondato, Edoardo Dighero, Alessia Democrito, Serena Losacco, Laura Emionite, Lucilla Nobbio, Irene Di Patrizi, Mattia Camera, Chiara Ghersi, Maddalena Ghelardoni, Francesco Lanfranchi, Francesca Vitale, Sonia Carta, Sabrina Chiesa, Carola Torazza, Marco Milanese, Matteo Bauckneht, Mehrnaz Hamedani, Federico Zaottini, Angelo Schenone, Carlo Martinoli, Federica Grillo, Gianmario Sambuceti

**Affiliations:** 1 Institute of BioImaging and Complex Biological Systems, National Research Council (CNR), 20054 Milan, Italy; 2 Nuclear Medicine, IRCCS Ospedale Policlinico San Martino di Genova, 16132 Genoa, Italy; 3 Nuclear Medicine, Department of Health Science, University of Genoa, 16132 Genoa, Italy; 4 Animal Facility, IRCCS Ospedale Policlinico San Martino di Genova, 16132 Genoa, Italy; 5 Clinica Neurologica Unit, IRCCS Ospedale Policlinico San Martino di Genova, 16132 Genoa, Italy; 6 Radiology Unit, IRCCS Ospedale Policlinico San Martino di Genova, 16132 Genoa, Italy; irene.dipatrizi@gmail.com; 7 DINOGMI, University of Genova, 16132 Genoa, Italy; 8 Pharmacology, Department of Pharmacy, University of Genoa, 16132 Genoa, Italy; 9 Radiology, Department of Health Science, University of Genoa, 16132 Genoa, Italy; 10 Pathology Unit, IRCCS Ospedale Policlinico San Martino di Genova, 16132 Genoa, Italy

**Keywords:** [^18^F]DPA-714, TSPO, PET, ALS, skeletal muscle, brain

## Abstract

Purpose: The skeletal muscle has been proposed to contribute to the progressive loss of motor neurons typical of amyotrophic lateral sclerosis (ALS). However, this mechanism has not yet been clarified due to the lack of suitable imaging tools. Here, we aimed to verify whether PET imaging of the translocator protein 18 kDa (TSPO) can detect a muscular abnormality in an experimental model of ALS. Methods: In vivo biodistribution and kinetics of [^18^F]DPA-714 were analyzed in skeletal muscle and brain of SOD1^G93A^ transgenic mice and in wildtype (WT) littermates. Both cohorts were divided into three groups (n = 6 each) to be studied at 60, 90 and 120 days. After microPET imaging, animals were sacrificed to evaluate inflammatory infiltrates by hematoxylin/eosin staining and TSPO expression by immunohistochemistry and Western blot in both quadriceps and brain. Results: [^18^F]DPA-714 uptake was higher in the skeletal muscles of SOD1^G93A^ than in WT mice in the preclinical phase (60 and 90 days) and further increased up to the symptomatic late stage (120 days). Inflammatory cells were absent in the quadriceps of SOD1^G93A^ mice whose myocytes, instead, showed a progressive increase in TSPO expression with advancing age. By contrast, brain tracer uptake and TSPO expression were comparably low in both groups, regardless of age and genotype. Conclusion: Upregulation of TSPO expression is characteristic of skeletal muscle, but not the brain, in the experimental SOD1^G93A^ mouse model of ALS. Tracers targeting this pathway have been mostly proposed for the evaluation of inflammatory processes within the central nervous system. Nevertheless, the ubiquitous nature of TSPO expression and its responsiveness to various signals may broaden the diagnostic potential of these tracers to include disease conditions beyond inflammation.

## 1. Introduction

For a long time, amyotrophic lateral sclerosis (ALS) has been almost exclusively attributed to the degeneration of upper and lower motor neurons (MNs), with skeletal muscle damage being considered as the innocent bystander of their progressive loss [1,2,3,4]. In recent years, several studies have configured MNs and skeletal muscle as a combined “neuromuscular unit”, with the effector component playing a relevant role in disease onset and progression [2].

Although the mechanisms underlying the ALS-associated impairment of the neuromuscular unit are still largely undefined, the introduction of positron-emitting tracers targeting the translocator protein 18 kDa (TSPO) permitted the detection of the presence of reactive gliosis and inflammation in the motor cortex of ALS patients [5,6,7,8] and in the brainstem of the SOD1^G93A^ mice as an experimental ALS model [9]. Initially known as the peripheral-type benzodiazepine receptor [10,11] prevalently expressed by inflammatory cells, TSPO has been found to be almost ubiquitous [12]. Measurable protein levels have been found in the skeletal muscle [13,14]. By contrast, TSPO abundance is very low in the central nervous system (CNS) [13,14], where local injury or inflammation upregulate its expression in astrocytes and microglia [6,15,16,17,18].

Due to its docking to the outer mitochondrial membrane, TSPO significantly contributes to the modulation of respiratory function and oxidative stress [19], as well as cholesterol binding [20], apoptosis [21], cell proliferation, and immune function [22]. These considerations indicate that skeletal muscle TSPO expression might be altered by ALS due to the high OXPHOS rate of this tissue and its early impairment by oxidative damage in experimental disease models [23].

Testing this hypothesis, here we show a detectable upregulation of TSPO expression, independent of inflammatory infiltrates, in the skeletal muscle of SOD1^G93A^ mice.

## 2. Materials and Methods

### 2.1. Radiochemistry

The synthesis of [^18^F]DPA-714 was performed using the GMP-compliant process designed on Trasis AllinOne 36 valves equipped with a semipreparative HPLC (Ans, Belgium). The procedure previously published by Cybulska et al. [24] was selected due to the availability of pharma-grade reagents.

Reagents and solvents were purchased from ABX (Rademberg, Germany), and cassettes and other materials were purchased from Trasis (Ans, Belgium) and BBraun (Melsungen, Germany). The GMP grade precursor tosylated DPA-714 was purchased from Pharmasynth (Tallinn, Estonia). The synthesis module Trasis AllinOne 36 was equipped with an Xbridge BEH C18 OBD, Waters (Etten-Leur, The Netherlands), and a UV detector set at 254 nm for real-time semipreparative purification. Sep-Pak C18 Plus cartridges were purchased from Waters Corporation (Etten-Leur, The Netherlands), and sterilizing filters from Cathivex-GV terminal filter (Merk-Millipore Ltd., Cork, Ireland, USA). ^18^F-fluorine solution was produced on-site using a Siemens Eclipse HP cyclotron (Erlangen, Germany).

DPA-714 was labeled with ^18^F at the 2-fluoroethyl moiety of the tosylated DPA-714, via SN2 nucleophilic substitution. The process involved the activation of ^18^F as K[^18^F]F Kryptofix-222 and its reaction in a “one pot” procedure with the tosyloxy precursor. Optimal labeling conditions were found using 4.5–5.5 mg (8.2–10.0 mmol) of precursor in anhydrous acetonitrile, reacting at 95 °C for 10 min. To remove most of both radioactive and cold by-products, the crude reaction mixture was first purified by an HPLC semipreparative system at a flow rate of 4 mL/min using isocratic elution with 0.1 M ammonium acetate/ethanol (55/45% *v*/*v*%) with a retention time of 10 ± 0.5 min. The collected radioactive fraction (5 ± 0.5 mL) underwent a second purification: after dilution with saline, [^18^F]DPA-714 was trapped on a C18 chromatographic cartridge and then recovered using ethanol 70%. [^18^F]DPA-714 was then eluted and sterilized by terminal filtration with a Cathivex GV filter.

The entire procedure lasted approximately 45 min. A full set of quality controls—as reported in the general monographs of the European Pharmacopoeia and the available literature—was carried out both for synthesis optimization (validation batches) and for all subsequent preparations. Chemical and radiochemical purity of the [^18^F]DPA-714 solution were determined in compliance with ICH Guideline Q2(R1) Validation of Analytical Procedures and European Pharmacopoeia Chromatographic Separation Techniques [25,26].

Radiosynthesis resulted in batches typically of 5–7 GBq of [^18^F]DPA-714, starting from 20 to 35 GBq of ^18^F, in 15 mL of final volume with overall radiochemical yield ranging from 17.4 to 22.6% (n = 15). Quality control results complied with the requirements of the European Pharmacopoeia up to the tracer expiry time of 6 h. All preparations resulted in a high [^18^F]DPA-714 radiochemical purity (ranging 98.6–99.0%), a high specific activity (364–613 GBq/μmol), and an acceptably low ethanol content (ranging 5.5–6.5%*v/v*%) [27].

### 2.2. Animal Models

B6SJL-Tg 1Gur/J SOD1^G93A^ mice were purchased from The Jackson Laboratory and were housed under a standard light/dark (12:12 h) cycle. Animals were provided with food and water ad libitum in the SPF Animal Facility of IRCCS Ospedale Policlinico San Martino, Genova, Italy. Heterozygous transgenic mice were obtained by mating heterozygous transgenic males with wildtype (WT) females (B6SJL). Transgenic mice were identified by PCR assay on DNA extracted from the tail. The primer sequences used for genotyping SOD1^G93A^ transgenic mice were as follows: 5′-CAT CAG CCC TAA TCC ATC TGA-3′, 5′-CGC GAC TAA CAA TCA AAG TGA-3′ (according to The Jackson Laboratory protocol).

Analysis was performed at three time points: at day #60 and day #90 (asymptomatic stage) and at day #120, after the appearance of motor impairment. For each age, six animals were evaluated for both SOD1^G93A^ and WT genotypes, accounting for 18 SOD1^G93A^ and 18 WT animals.

All procedures were performed in accordance with Italian 26/2014 and EU 2010/63/UE directives. In particular, the research protocols presented in this study were conducted in accordance with the ARRIVE guidelines and were approved by the IRCCS Ospedale Policlinico San Martino OPBA (Institutional Animal Welfare Body) as well as by the Italian Ministry of Health (project number approval 301/2023-PR, 2023-04-11). During late-stage disease, access to food and water were provided by adding a Petri dish full of water on the floor of the cage and by using a water dispenser with a long spout.

### 2.3. Experimental Micro-PET Scanning and Image Analysis

For micro-PET imaging, mice were weighed and anesthetized with intra-peritoneal ketamine (100 mg/kg) and xylazine (10 mg/kg). Mice were positioned on the bed of a dedicated micro-PET system (Albira, Bruker, USA) whose dual ring configuration allows the acquisition of the whole mouse body. [^18^F]DPA-714 (7–10 MBq) was injected through a tail vein soon after the start of a dynamic acquisition lasting 45 min, based on previous studies documenting a fast uptake of this tracer in the brain of rodents [28,29]. The list-mode acquisition was binned according to the following frame sequence: 18 frames × 10 s; 2 frames × 60 s; and 8 frames × 300 s. Tracer was manually injected. Images were reconstructed using the maximal likelihood expectation maximization method (MLEM), using the scanner proprietary software.

Obtained images were qualitatively inspected. Therefore, two nuclear physicians expert in mouse imaging and unaware of the model nature (transgenic or wildtype) manually drew volumes of interest (VOIs) on the lungs, the brain, the spleen and the skeletal muscle of the thighs using the routine of PMOD software version 3.405 (PMOD, Bruker, Zurich, Switzerland) according to our standard procedure [23,30]. The software used for this proFinal radioactivity concentration was expressed as a standardized uptake value (SUV) according to the following formula:

$$SUV=\frac{\left[DPA-714\right]\times body\;weight}{Injected\;dose}$$

where [DPA-714] indicates the local tracer concentration (KBq/mL); body weight is expressed in grams and injected dose is expressed in KBq.

### 2.4. Ex Vivo Studies

The day after image acquisition, euthanasia was performed by CO_2_ asphyxiation when mice failed to right themselves within 30 s of being placed on their side (humane endpoint) [31]. The brain, quadriceps and spleen were isolated for subsequent evaluations.

Western blot: For Western blot (WB) experiments, proteins were extracted from the homogenates of brain cortex, spleen, and skeletal muscles using RIPA buffer associated with protease inhibitor cocktail. Protein lysates were separated by SDS-PAGE and transferred to PVDF membranes (Immobilon-P, Millipore Spa). Thereafter, they were incubated with the following antibodies: anti-TSPO monoclonal antibody 4H2 (Product # MA5-24844, dilution 1:2000) from Invitrogen, anti-Rabbit IgG HPR-Linked Antibody (Product #7074, dilution 1:10.000) and Anti-Mouse IgG HRP (Product #7076, dilution 1:10.000) from Cell Signaling Technology (Danvers, MA 01915, USA) Anti-GAPDH (glyceraldehyde 3-phosphate dehydrogenase) 2D4A7 (Product # NB300-328H, dilution 1:10.000) from Novus Biologicals. For spleen analysis, the selected housekeeping protein was anti-β-Actin (Product #A5316, dilution 1:10.000; Sigma-Aldrich, Saint Louis, MO 63103, USA), in order to overcome the weak GAPDH signal. Band intensities were quantified using NineAlliance software Q9 (UVITEC, Cambridge, UK). The original images are reported in Supplementary Figure S1.

Histology: Quadriceps were fixed in 10% buffered formalin for a standard time of 24 h at 4 °C. They were sectioned perpendicularly to the muscle fibers and routinely processed overnight in automated processors. All formalin-fixed paraffin-embedded tissues from both control and SOD1^G93A^ mice were sectioned using a microtome, and 4-micron-thick unstained sections were placed on uncharged slides for hematoxylin and eosin staining.

Immunohistochemistry: For the immunohistochemistry, 4-micron-thick unstained sections were cut and placed on Superfrost Plus (Thermo Scientific, Braunschweig, Germany) slides. Staining for TSPO and CD68 (Abcam, Cambridge, UK, and Diagnostic BioSystems, CA, USA, respectively) was performed manually according to manufacturers’ instructions. Tissue sections were deparaffinized and rehydrated. Antigen retrieval was carried out by microwaving (30 min at 100 Watts) in a citrate buffer at pH 6. Endogenous peroxidases were blocked with 5% H_2_O_2_ for 10 min. Reactions were developed using the DAKO EnVision + System-HRP labeled polymer anti-Rabbit coupled with a DAB detection kit (DAKO North America Inc., Carpinteria, CA, USA). The slides of muscle samples were then counterstained with hematoxylin and cover slipped. Reactions were evaluated based on the extent and intensity of cytoplasmic expression.

### 2.5. Autoradiography

The distribution of [^18^F]DPA-714 binding sites—and thus of TSPO expression of quadriceps—was assessed by in vitro autoradiography. One quadricep sample was analyzed in each group. After the same procedure used for hematoxylin/eosin staining, paraffin-embedded tissue sections were put into xylene overnight and, again, three times for 15 min the next day. Thereafter, three 15 min exposures were applied with the slices’ immersion in distilled water containing 95%, 70% and 50% ethanol. Finally, for target retrieval, slices were submitted to a 40 min heating period at 60 °C in a 0.1 M citrate buffer (pH 6.0). All slides were simultaneously incubated for 30 min in saline containing 200 KBq/mL [^18^F]DPA-714 and, thus, washed twice with saline. Thereafter, air-dried slides were exposed to a laser scanner Cyclone^®^ Plus Storage Phosphor System (Perkin Elmer, inc.) and subsequently scanned. Count densities (photostimulated luminescence per unit area, PSL/mm^2^) were represented according to the routine of the imageJ platform version 1.54 (Fiji, NHI, US), while no attempt was made to estimate absolute radioactivity content in any sample.

### 2.6. Statistical Analysis

All data are reported as mean ± standard deviation. The effect of age and genotype on the studied variable was tested by two-way ANOVA. The number of analyzed samples was always ≥3 and is reported in each corresponding figure. All statistical analyses were carried out using a dedicated software package, GraphPad Prism version 8 (GraphPad, San Diego, CA, USA).

## 3. Results

### 3.1. [^18^F]DPA-714 Uptake

In agreement with previous experience [32], SOD1^G93A^ mice showed a normal motor function up to 90 days, with the evident motor impairment appearing thereafter. At all studied ages, body weight was slightly, yet not significantly, lower in transgenic mice with respect to WT littermates.

**Figure 1:**
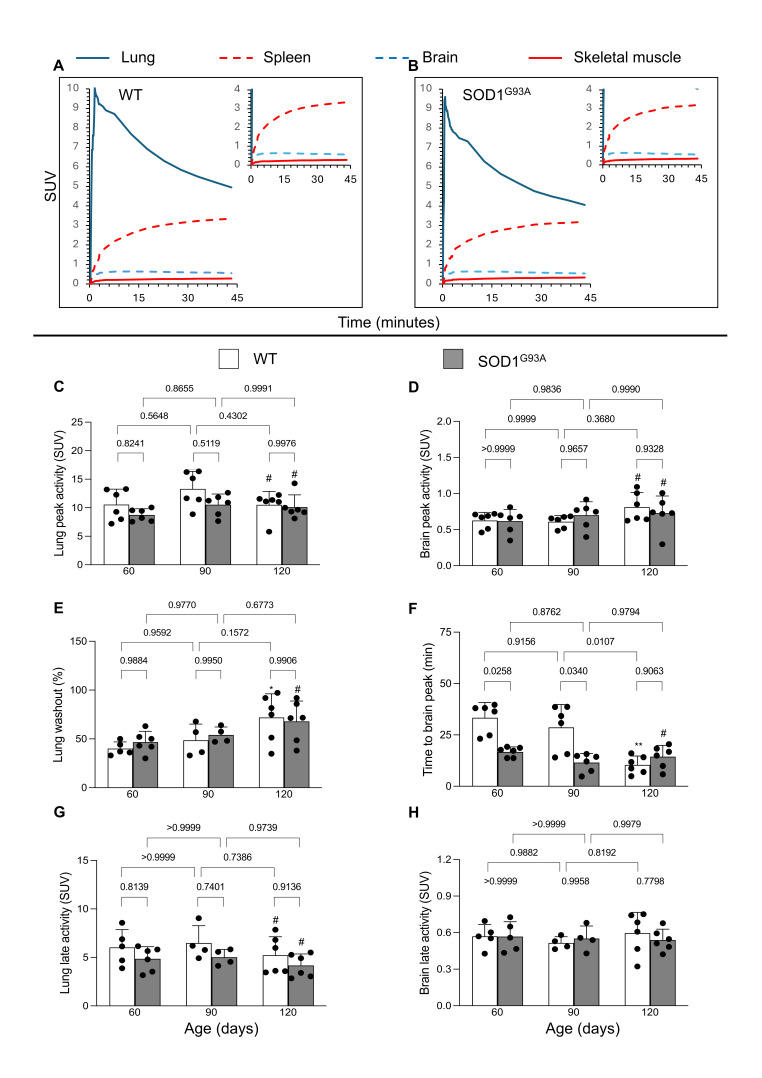
[^18^F]DPA-714 kinetics at dynamic micro-PET imaging. [^18^F]DPA-714 time–concentration curves in different organs of (**A**) WT and (**B**) SOD1^G93A^ mice. [^18^F]DPA-714 kinetics in WT (white) and SOD1^G93A^ (gray) mice at different ages: lung and brain (**C**,**D**) peak activity, (**E**,**F**) washout rate, and (**G**,**H**) final SUV. Statistical significance (*p* values) is reported over the brackets. The comparisons between the third and first evaluation time points are instead reported as follows: * *p* = 0.025 and ** *p* = 0.0036 and # *p* > 0.8 vs. the corresponding group at 60 days.

At dynamic micro-PET imaging, [^18^F]DPA-714 time–concentration curves showed a markedly different behavior in the studied organs, with high early uptake in the lungs as opposed to lower values in the brain, spleen and skeletal muscle (Figure 1A,B).

Lung tracer concentration peaked at approximately three minutes after injection in all groups to a maximal SUV that was independent of age and genotype (Figure 1C). Thereafter, lung radioactivity showed an evident washout whose rate progressively increased with advancing age in both groups (*p* < 0.001, Figure 1E). The final SUV was independent of age, while it was slightly, but not significantly, lower in the SOD1^G93A^ with respect to the WT mice at all time points (Figure 1G).

Brain time–concentration curves showed a largely different pattern. The peak of radioactivity concentration was similar in both groups at all studied ages (Figure 1D). By contrast, the time needed to reach this value was longer in WT than in SOD1^G93A^ mice aged 60 or 90 days (*p* < 0.05, Figure 1F). In the oldest models, however, this difference disappeared due to the acceleration of tracer uptake in WT mice. Thus, late tracer retention was remarkably similar in the two models and independent of age (Figure 1H).

Spleen uptake progressively increased, and no washout could be documented, at least until the latest imaging time (Figure 1A,B).

**Figure 2:**
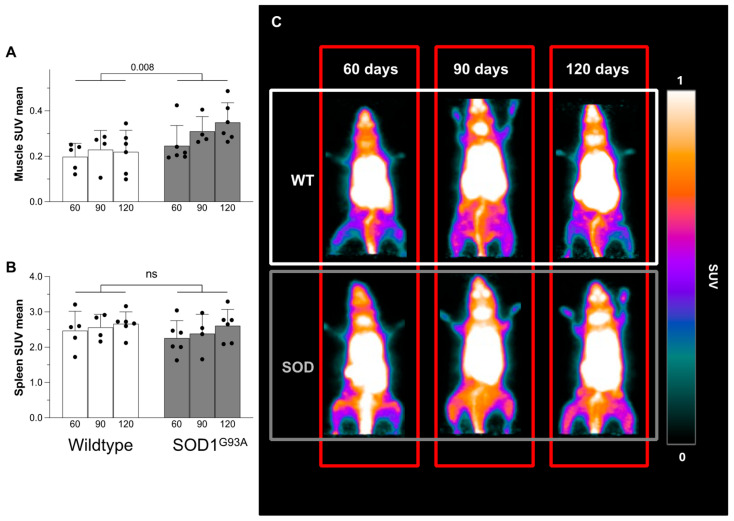
[^18^F]DPA-714 uptake at micro-PET imaging in WT (white) and SOD1^G93A^ (gray) mice at different ages. Skeletal muscle (**A**) and spleen (**B**) final uptake. (**C**) Representative micro-PET images of WT and SOD1^G93A^ mice. Statistical significance (*p* values) is reported over the brackets.

Tracer uptake kinetics in the skeletal muscle reproduced the pattern observed in the spleen and showed a progressive increase throughout the imaging time (Figure 1A,B). The final SUV in this district was higher in SOD1^G93A^ mice with respect to their wildtype littermates (Figure 2A,C). Moreover, [^18^F]DPA-714 uptake progressively increased in the ALS murine model as opposed to an evident stability in the control group (Figure 2A,C and Supplementary Video File). This pattern did not characterize the spleen, whose SUV mean was independent of genotype (Figure 2B).

### 3.2. Overexpression of TSPO in Skeletal Muscle of Transgenic Mice

**Figure 3:**
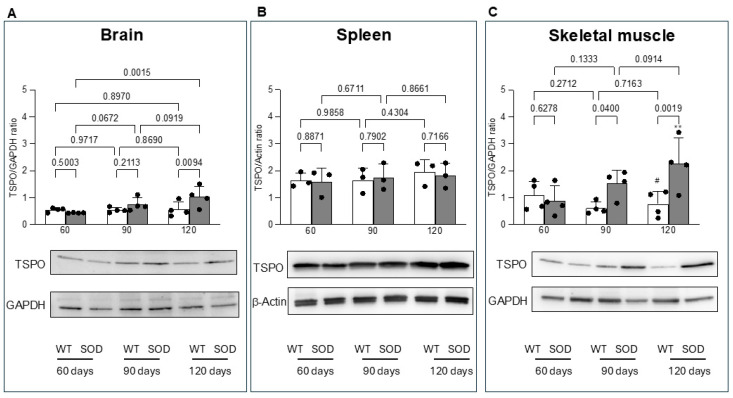
Western blot analysis. TSPO/GAPDH ratio in brain (**A**) and skeletal muscle (**C**) as well as TSPO/β-Actin ratio (**B**) in spleen of WT and SOD1^G93A^ mice at different ages. Statistical significance (*p* values) is reported over the brackets. The comparisons between the third and the first evaluation for the skeletal muscle are instead reported as follows: # *p* > 0.8 and ** *p* = 0.004 vs. corresponding group at 60 days.

The Western blot analysis agreed with the data provided by microPET imaging. TSPO abundance in the brain was similar in WT and asymptomatic SOD1^G93A^ mice (60–90 days of life), Figure 3A. However, protein expression showed a progressive increase that became significantly higher in SOD1^G93A^ mice than in WT ones at 120 days (Figure 3A). By contrast, the TSPO abundance of the spleen was comparably represented in both genotypes at all ages (Figure 3B). Finally, quadriceps showed a different behavior. TSPO expression was similar in the two groups 60 days after birth (Figure 3C). However, it progressively increased at 90 and 120 days in SOD1^G93A^ mice, while remaining substantially unchanged in control ones (Figure 3C).

### 3.3. Histology and Immunohistochemistry of Skeletal Muscle

**Figure 4:**
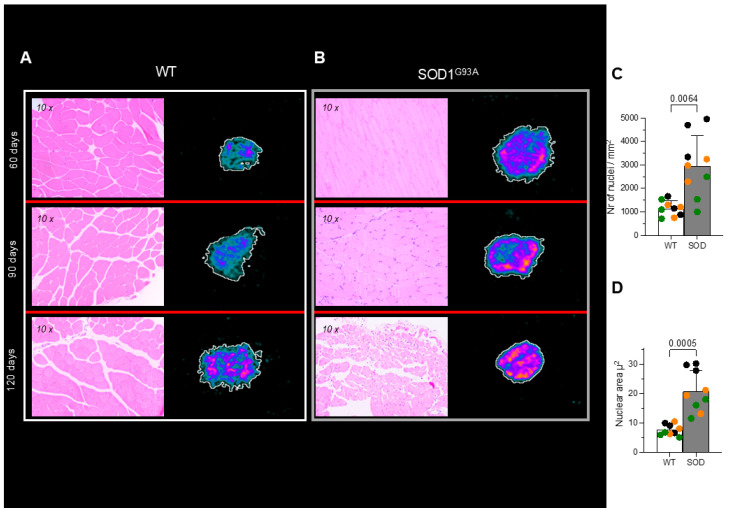
Histology (10x). Hematoxylin/eosin staining of skeletal muscle tissue from the quadriceps of (**A**) WT and (**B**) SOD1^G93A^ mice at different ages, showing no inflammatory infiltrates but revealing a progressive atrophy of myofibers in the SOD1^G93A^ group. In the right side of each panel, the autoradiography of the corresponding model is reported. (**C**) Number of nuclei and (**D**) nuclear area in WT and SOD1^G93A^ skeletal muscle tissues. In panels C and D, data are reported for wildtype (WT) (white) and SOD1^G93A^ (gray), with age classified according to the following color code: green = 60 days, orange = 90 days and black = 120 days. Statistical significance (*p*-values) is reported over the brackets.

**Figure 5:**
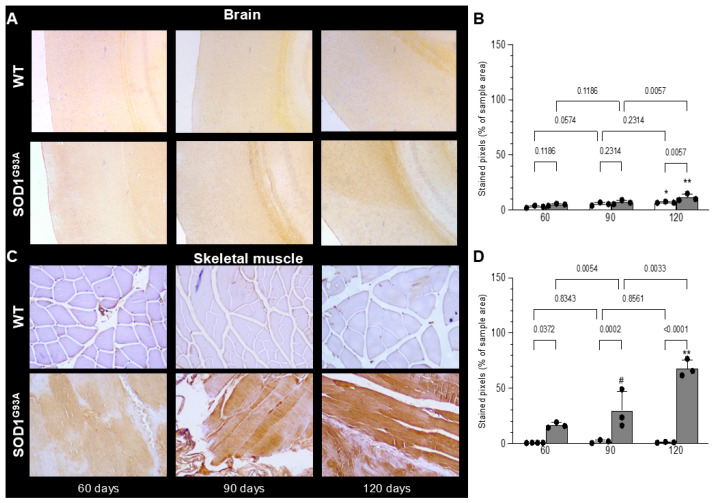
TSPO immunostaining of brains (**A**, magnification ×40) and skeletal muscle tissues (**C**, magnification ×200) from WT (white) and SOD1^G93A^ mice (gray columns) at different ages. Immunostaining was very faint in brain samples. In quadriceps specimens, staining was absent in WT mice, while it progressively increased in the SOD1^G93A^ ones. No inflammatory infiltration was detectable. Quantitative analysis of prevalence of stained pixels is reported in the corresponding right panels (**B**,**D**). Statistical significance (*p*-values) is reported over the brackets and indicates that age increased TSPO immunoreactivity in both the brain and skeletal muscle of SOD mice as opposed to an apparently not significant response in WT ones. The comparisons between the third and first evaluation time points are instead reported as follows: (# *p* < 0.8, * *p* = 0.036, ** *p* < 0.0001 vs. corresponding group at 60 days).

Hematoxylin/eosin staining showed the absence of appreciable inflammatory infiltrates in the quadriceps of both WT littermates and SOD1^G93A^ mice (Figure 4A,B). By contrast, it highlighted a progressive atrophy of myofibers in transgenic mice with respect to control ones (Figure 4A,B). Nuclei of mutated skeletal muscle appeared more numerous (Figure 4C), enlarged (Figure 4D), and closer together, probably due to the tissue degeneration. Furthermore, muscle atrophy and nuclear modifications worsened with age in SOD1^G93A^ mice while remaining invariant in their WT littermates (Figure 4C,D). Autoradiography was performed to evaluate the tracer distribution pattern, and no attempt was put forward to estimate tracer concentration, considering the availability of PET data. These experiments showed a homogeneous tracer uptake throughout the muscular slice under all conditions (Figure 4A,B). This pattern was reproduced by the distribution of TSPO expression assayed by immunohistochemistry, as reported in Figure 5.

In agreement with data reported by Gargiulo et al. [9], TSPO positivity was very low in the motor cortex of WT brains and showed a modest, though significant, increase at 120 days (Figure 5A,B). A similar trend also occurred in SOD1^G93A^ mice that showed a slightly higher prevalence of TSPO immunoreacting pixels at the oldest age with the appearance of an evident motor impairment (Figure 5A,B).

When skeletal muscles were analyzed, staining was virtually absent in all control mice (Figure 5C,D). By contrast, in SOD1^G93A^ mice, it was initially faint to moderate and heterogeneous at 60 days of age; it became more intense and widespread (but still heterogeneous) at 90 days and reached its maximum intensity in 120-day-old mice (Figure 5C,D).

**Figure 6:**
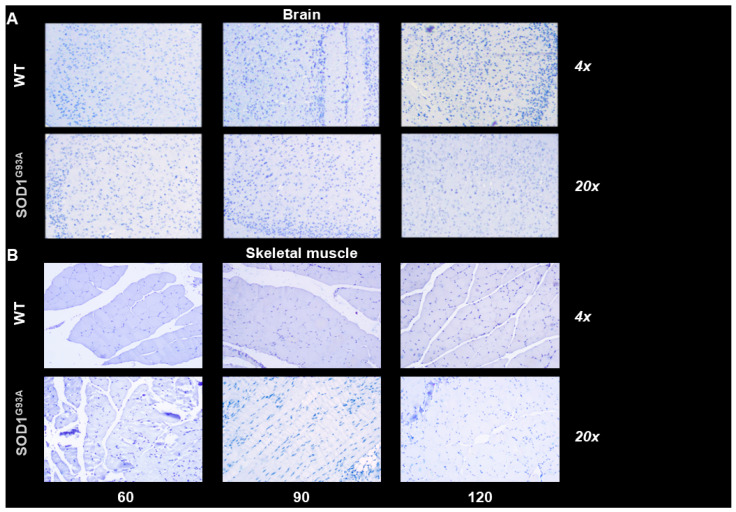
Immunostaining for CD68 of brains (**A**) and skeletal muscle slices (**B**) from WT (white) and SOD1^G93A^ mice (gray). No brown area, indicative of anti-CD68 antibody, could be detected in both tissues.

The direct involvement of myocytes in the enhanced expression of TSPO caused by SOD mutation agreed with the homogeneous distribution of [^18^F]DPA-714 radioactivity observed at the autoradiographic visualization of in vitro tracer uptake in both groups (Figure 4A,B). Finally, the absence of inflammatory infiltrates was confirmed by CD68 staining since this marker of both macrophages and activated microglia [33] was not detected in the brain or in the skeletal muscle of WT and SOD1^G93A^ mice (Figure 6A,B).

## 4. Discussion

Progressive motor impairment is the main characteristic of ALS. Although this behavior has been attributed to motor neuron damage, recent evidence suggests an active role of skeletal muscle in the pathogenesis of the disease. Our experimental data are consistent with this concept and support a selective cell-autonomous degeneration in the skeletal muscle, less (or later) represented in the brain, extending models proposed for other neurodegenerative diseases leading to a motor impairment [34]. This feature is reproduced by both the TSPO expression and the uptake of its targeted radioligand [^18^F]DPA-714. Although the present data do not conclusively identify binding to TSPO as the mechanism underlying the increased tracer uptake, our findings demonstrate that the skeletal muscle of SOD1^G93A^ mice exhibits increased radioactivity accumulation following [^18^F]DPA-714 administration, as observed through microPET imaging. This finding matched the TSPO expression, as defined by both immunohistochemistry and WB. It selectively involved the myocytes since the homogeneous tracer distribution at autoradiography matched the absence of inflammatory infiltrates detected by either hematoxylin/eosin staining or CD68 immunohistochemical evaluation. Altogether, these data indicate that [^18^F]DPA-714 is not a selective marker of inflammation. Rather, the enhanced TSPO expression in myocytes seems to precede the documented inflammatory reaction of the brain cortex [31,35]. This finding agrees with the relatively low prevalence and size of inflammatory infiltrates that have been reported in the skeletal muscle of both SOD1G93 mice [36] and ALS patients [37].

### PET Evaluation of TSPO Expression

Although the mechanisms upregulating the TSPO expression are still not completely clarified, this protein contributes to regulating a variety of biochemical processes such as the mitochondrial calcium flux [38], the cholesterol transfer into mitochondria for promoting steroidogenesis, and response to redox stress [39]. In agreement with this multiple function, TSPO expression has been found to be modulated by several diseases in different tissues and organs [28], such as the liver [40], heart [41], endocrine glands [41], kidneys [42], pancreas [43] and lungs [44]. Similarly, a wide literature, recently synthesized by Wongso et al. [45], already reported a potential for TSPO-targeting radioligands for both diagnosis and treatment of various types of cancers, including colorectal, brain, breast, prostate, and lung cancers, as well as melanoma.

Time-activity curves were similar in the skeletal muscle and in the TSPO-rich splenic tissue as opposed to the dual-phase pattern observed in the lungs. More importantly, both WB and immunohistochemical evaluations documented a nice agreement between the enhanced [^18^F]DPA-714 uptake and the enhanced TSPO expression in the quadriceps of SOD1^G93A^ mice. The adherence between SUV and TSPO abundance was further confirmed by autoradiography that permitted the identification of the selectivity of tracer uptake despite the absence of contamination by its radioactive metabolites. Similarly, the direct involvement of myocytes in the increased TSPO expression was confirmed by both hematoxylin/eosin and CD68 staining that ruled out the presence of inflammatory infiltrations. Finally, the presence of a specific and primary damage to the skeletal muscle was confirmed by the increase in number and size of nuclei that configured an opposite pattern with respect to the muscular atrophy induced by denervation [46,47].

The link between tracer uptake and tissue metabolic pattern might apply to the central nervous tissue as well. Campanella’s group previously documented that TSPO overexpression is linked to the loss of mitophagy in a model of Parkinson’s disease [17]. This concept has been more recently confirmed by Magrì et al. who, studying the spinal cords of the same SOD1^G93A^ mice included in our study, observed that the increased TSPO levels were also associated with an impaired ATP-linked oxygen consumption rate by glial cells [15].

Our findings are consistent with previous studies, demonstrating that increased TSPO expression and enhanced [^18^F]DPA-714 uptake are not exclusive markers of inflammation but can also indicate a direct response of damaged tissue [12,40,41,42,43,44,45]. These data suggest a potential shift in the interpretation of PET imaging with TSPO-targeting tracers. Specifically, the lack of increased tracer uptake in the brains of SOD1^G93A^ mice corresponded with the limited TSPO expression in this region. Thus, our findings do not diminish the utility of TSPO-targeting tracers in evaluating neuroinflammation. Instead, they propose an expanded diagnostic potential for these tracers, extending their application to conditions characterized by elevated TSPO expression, regardless of the affected tissue or underlying disorder.

## 5. Conclusions

The main finding of the present study is the evidence of an increased [^18^F]DPA-714 uptake in the quadriceps of SOD1^G93A^ mice. This observation agrees with the upregulation of myocyte TSPO expression documented by both immunohistochemistry and WB experiments. The brain showed a relatively less evident increase in protein content that matched the modest, and not significant, enhancement in tracer retention. This finding only apparently disagrees with previous experience reporting an increased uptake of [^18^F]DPA-714 in the motor cortex of ALS patients. Indeed, most of these studies compared tracer kinetics in this cortical area with the corresponding features in other intracranial structures. This procedure permits the identification of abnormalities in tracer distribution but does not provide any insight into the effect of the disease on absolute uptake or distribution volume [7]. The spatial resolution of our microPET facility, coupled with the absence of CT/MRI anatomical pictures, prevented the possibility of selectively analyzing specific regions of the mouse motor cortex. However, the “normal” values of tracer uptake in the whole brain of SOD1^G93A^ mice agree with the data of Gargiulo and coworkers [9]. Indeed, in that study, [^18^F]DPA-714 retention was increased only in the brainstem and the cervical spinal cord of this experimental ALS model.

In conclusion, the increased TSPO expression observed in SOD1^G93A^ mice aligns with the presence of metabolic abnormalities selectively affecting the skeletal muscle of ALS patients [48,49,50]. This evidence suggests that the mechanisms influencing [^18^F]DPA-714 uptake differ fundamentally in nature and time course between striated musculature and the central nervous system. This divergence highlights the potential of PET imaging with tracers that specifically target TSPO to provide novel insights into the distinct pathophysiological mechanisms underlying the onset and progression of ALS.

The limited adherence of the selected experimental to the mechanisms underlying disease progression in the majority of ALS patients could not be tested in this experimental study. Nevertheless, obtained data might represent the basis for future studies aiming to verify whether the link between TSPO expression and [^18^F]DPA-714 uptake might represent an early biomarker of ALS or a potential tool for evaluating the effectiveness of future therapeutic strategies aimed at improving motor function and, ultimately, the quality of life for these patients.

## Data Availability

The original contributions presented in this study are included in the article/Supplementary Material. Further inquiries can be directed to the corresponding authors.

## Supplementary Materials

The following supporting information can be downloaded at https://www.mdpi.com/article/10.3390/biom15060799/s1, Figure S1: Original images of the Western blots; Video S1: [^18^F]DPA-714 uptake at micro-PET imaging in wild-type (WT) and SOD1^G93A^ (SODy) mice at different ages.

## References

[1] Mead R.J., Shan N., Reiser H.J., Marshall F., Shaw P.J. (2023). Amyotrophic lateral sclerosis: A neurodegenerative disorder poised for successful therapeutic translation. Nat. Rev. Drug Discov..

[2] Shefner J.M., Musaro A., Ngo S.T., Lunetta C., Steyn F.J., Robitaille R., De Carvalho M., Rutkove S., Ludolph A.C., Dupuis L. (2023). Skeletal muscle in amyotrophic lateral sclerosis. Brain.

[3] Pikatza-Menoio O., Elicegui A., Bengoetxea X., Naldaiz-Gastesi N., López de Munain A., Gerenu G., Gil-Bea F.J., Alonso-Martín S. (2021). The Skeletal Muscle Emerges as a New Disease Target in Amyotrophic Lateral Sclerosis. J. Pers. Med..

[4] Loeffler J.P., Picchiarelli G., Dupuis L., Gonzalez De Aguilar J.L. (2016). The Role of Skeletal Muscle in Amyotrophic Lateral Sclerosis. Brain Pathol..

[5] Gao C., Jiang J., Tan Y., Chen S. (2023). Microglia in neurodegenerative diseases: Mechanism and potential therapeutic targets. Signal Transduct. Target. Ther..

[6] Dimitrova-Shumkovska J., Krstanoski L., Veenman L. (2020). Diagnostic and Therapeutic Potential of TSPO Studies Regarding Neurodegenerative Diseases, Psychiatric Disorders, Alcohol Use Disorders, Traumatic Brain Injury, and Stroke: An Update. Cells.

[7] Van Weehaeghe D., Babu S., De Vocht J., Zürcher N.R., Chew S., Tseng C.J., Loggia M.L., Koole M., Rezaei A., Schramm G. (2020). Moving Toward Multicenter Therapeutic Trials in Amyotrophic Lateral Sclerosis: Feasibility of Data Pooling Using Different Translocator Protein PET Radioligands. J. Nucl. Med..

[8] Werry E.L., Bright F.M., Piguet O., Ittner L.M., Halliday G.M., Hodges J.R., Kiernan M.C., Loy C.T., Kril J.J., Kassiou M. (2019). Recent Developments in TSPO PET Imaging as A Biomarker of Neuroinflammation in Neurodegenerative Disorders. Int. J. Mol. Sci..

[9] Gargiulo S., Anzilotti S., Coda A.R., Gramanzini M., Greco A., Panico M., Vinciguerra A., Zannetti A., Vicidomini C., Dollé F. (2016). Imaging of brain TSPO expression in a mouse model of amyotrophic lateral sclerosis with ^18^F-DPA-714 and micro-PET/CT. Eur. J. Nucl. Med. Mol. Imaging.

[10] Politis M., Su P., Piccini P. (2012). Imaging of microglia in patients with neurodegenerative disorders. Front. Pharmacol..

[11] Papadopoulos V., Baraldi M., Guilarte T.R., Knudsen T.B., Lacapère J.J., Lindemann P., Norenberg M.D., Nutt D., Weizman A., Zhang M.R. (2006). Translocator protein (18 kDa): New nomenclature for the peripheral-type benzodiazepine receptor based on its structure and molecular function. Trends Pharmacol. Sci..

[12] Barresi E., Robello M., Costa B., Da Pozzo E., Baglini E., Salerno S., Da Settimo F., Martini C., Taliani S. (2021). An update into the medicinal chemistry of translocator protein (TSPO) ligands. Eur. J. Med. Chem..

[13] Wang H.J., Fan J., Papadopoulos V. (2012). Translocator protein (Tspo) gene promoter-driven green fluorescent protein synthesis in transgenic mice: An in vivo model to study Tspo transcription. Cell Tissue Res..

[14] Fan J., Rone M.B., Papadopoulos V. (2009). Translocator protein 2 is involved in cholesterol redistribution during erythropoiesis. J. Biol. Chem..

[15] Magrì A., Lipari C.L.R., Risiglione P., Zimbone S., Guarino F., Caccamo A., Messina A. (2023). ERK1/2-dependent TSPO overactivation associates with the loss of mitophagy and mitochondrial respiration in ALS. Cell Death Dis..

[16] Guilarte T.R., Rodichkin A.N., McGlothan J.L., Acanda De La Rocha A.M., Azzam D.J. (2022). Imaging neuroinflammation with TSPO: A new perspective on the cellular sources and subcellular localization. Pharmacol. Ther..

[17] Frison M., Faccenda D., Abeti R., Rigon M., Strobbe D., England-Rendon B.S., Cash D., Barnes K., Sadeghian M., Sajic M. (2021). The translocator protein (TSPO) is prodromal to mitophagy loss in neurotoxicity. Mol. Psychiatry.

[18] Harberts E., Datta D., Chen S., Wohler J.E., Oh U., Jacobson S. (2013). Translocator protein 18 kDa (TSPO) expression in multiple sclerosis patients. J. Neuroimmune Pharmacol..

[19] Batoko H., Veljanovski V., Jurkiewicz P. (2015). Enigmatic Translocator protein (TSPO) and cellular stress regulation. Trends Biochem. Sci..

[20] Jaipuria G., Leonov A., Giller K., Vasa S.K., Jaremko Ł., Jaremko M., Linser R., Becker S., Zweckstetter M. (2017). Cholesterol-mediated allosteric regulation of the mitochondrial translocator protein structure. Nat. Commun..

[21] Pan J.H., Kang Y.Q., Li Q., Xing W., Chen Y.H., Yan Y., Luo D.X., Qiu Y., Yuan Y.F., Zeng W.A. (2023). TSPO is a novel biomarker for prognosis that regulates cell proliferation through influencing mitochondrial functions in HCC. Heliyon.

[22] Betlazar C., Middleton R.J., Banati R., Liu G.J. (2020). The Translocator Protein (TSPO) in Mitochondrial Bioenergetics and Immune Processes. Cells.

[23] Marini C., Cossu V., Bonifacino T., Bauckneht M., Torazza C., Bruno S., Castellani P., Ravera S., Milanese M., Venturi C. (2020). Mechanisms underlying the predictive power of high skeletal muscle uptake of FDG in amyotrophic lateral sclerosis. EJNMMI Res..

[24] Cybulska K.A., Bloemers V., Perk L.R., Laverman P. (2021). Optimised GMP-compliant production of [^18^F]DPA-714 on the Trasis AllinOne module. EJNMMI Radiopharm. Chem..

[25] (2014). CPMP/ICH/381/95—ICH Harmonised Tripartite Guideline—Validation of Analytical Procedures: Text and Methodology Q2(R1). https://www.ema.europa.eu/en/ich-q2-r1-validation-analytical-procedures-text-methodology.

[26] (2016). European Pharmacopoeia 9.5, 2.2.46 Chromatographic Separation Techniques (07/2016:20246).

[27] Saba W., Goutal S., Auvity S., Kuhnast B., Coulon C., Kouyoumdjian V., Buvat I., Leroy C., Tournier N. (2018). Imaging the neuroimmune response to alcohol exposure in adolescent baboons: A TSPO PET study using ^18^F-DPA-714. Addict. Biol..

[28] Vicidomini C., Panico M., Greco A., Gargiulo S., Coda A.R., Zannetti A., Gramanzini M., Roviello G.N., Quarantelli M., Alfano B. (2015). In vivo imaging and characterization of [^18^F]DPA-714, a potential new TSPO ligand, in mouse brain and peripheral tissues using small-animal PET. Nucl. Med. Biol..

[29] Martín A., Boisgard R., Thézé B., Van Camp N., Kuhnast B., Damont A., Kassiou M., Dollé F., Tavitian B. (2010). Evaluation of the PBR/TSPO radioligand [^18^F]DPA-714 in a rat model of focal cerebral ischemia. J. Cereb. Blood Flow Metab..

[30] Bauckneht M., Cossu V., Castellani P., Piccioli P., Orengo A.M., Emionite L., Di Giulio F., Donegani M.I., Miceli A., Raffa S. (2020). FDG uptake tracks the oxidative damage in diabetic skeletal muscle: An experimental study. Mol. Metab..

[31] Van Weehaeghe D., Van Schoor E., De Vocht J., Koole M., Attili B., Celen S., Declercq L., Thal D.R., Van Damme P., Bormans G. (2020). TSPO Versus P2X7 as a Target for Neuroinflammation: An In Vitro and In Vivo Study. J. Nucl. Med..

[32] Hegedus J., Putman C.T., Tyreman N., Gordon T. (2008). Preferential motor unit loss in the SOD1 G93A transgenic mouse model of amyotrophic lateral sclerosis. J. Physiol..

[33] Kovacs G.G. (2017). Cellular reactions of the central nervous system. Handb. Clin. Neurol..

[34] Romer S.H., Metzger S., Peraza K., Wright M.C., Jobe D.S., Song L.S., Rich M.M., Foy B.D., Talmadge R.J., Voss A.A. (2021). A mouse model of Huntington’s disease shows altered ultrastructure of transverse tubules in skeletal muscle fibers. J. Gen. Physiol..

[35] Corcia P., Tauber C., Vercoullie J., Arlicot N., Prunier C., Praline J., Nicolas G., Venel Y., Hommet C., Baulieu J.L. (2012). Molecular imaging of microglial activation in amyotrophic lateral sclerosis. PLoS ONE.

[36] Chiu I.M., Phatnani H., Kuligowski M., Tapia J.C., Carrasco M.A., Zhang M., Maniatis T., Carroll M.C. (2009). Activation of innate and humoral immunity in the peripheral nervous system of ALS transgenic mice. Proc. Natl. Acad. Sci. USA.

[37] Al-Sarraj S., King A., Cleveland M., Pradat P.F., Corse A., Rothstein J.D., Leigh P.N., Abila B., Bates S., Wurthner J. (2014). Mitochondrial abnormalities and low grade inflammation are present in the skeletal muscle of a minority of patients with amyotrophic lateral sclerosis; an observational myopathology study. Acta Neuropathol. Commun..

[38] Gatliff J., East D.A., Singh A., Alvarez M.S., Frison M., Matic I., Ferraina C., Sampson N., Turkheimer F., Campanella M. (2017). A role for TSPO in mitochondrial Ca^2+^ homeostasis and redox stress signaling. Cell Death Dis..

[39] Carayon P., Portier M., Dussossoy D., Bord A., Petitprêtre G., Canat X., Le Fur G., Casellas P. (1996). Involvement of peripheral benzodiazepine receptors in the protection of hematopoietic cells against oxygen radical damage. Blood.

[40] Li Y., Chen L., Papadopoulos V. (2023). The mitochondrial translocator protein (TSPO, 18 kDa): A key multifunctional molecule in liver diseases. Biochimie.

[41] James M.L., Fulton R.R., Vercoullie J., Henderson D.J., Garreau L., Chalon S., Dolle F., Costa B., Guilloteau D., Kassiou M. (2008). DPA-714, a new translocator protein-specific ligand: Synthesis, radiofluorination, and pharmacologic characterization. J. Nucl. Med..

[42] Thuillier R., Hauet T. (2012). Role of translocator protein in renal ischemia reperfusion, renal preservation and acute kidney injury. Curr. Mol. Med..

[43] Guillemain G., Khemtemourian L., Brehat J., Morin D., Movassat J., Tourrel-Cuzin C., Lacapere J.J. (2024). TSPO in pancreatic beta cells and its possible involvement in type 2 diabetes. Biochimie.

[44] Zhang C., Sheng M., Lv J., Cao Y., Chen D., Jia L., Sun Y., Ren Y., Li L., Weng Y. (2023). Single-cell analysis reveals the immune heterogeneity and interactions in lungs undergoing hepatic ischemia-reperfusion. Int. Immunopharmacol..

[45] Wongso H., Kurniawan A., Setiadi Y., Kusumaningrum C.E., Widyasari E.M., Wibawa T.H.A., Mahendra I., Febrian M.B., Sriyani M.E., Halimah I. (2024). Translocator Protein 18 kDa (TSPO): A Promising Molecular Target for Image-Guided Surgery of Solid Cancers. Adv. Pharm. Bull..

[46] Gundersen K., Bruusgaard J.C. (2008). Nuclear domains during muscle atrophy: Nuclei lost or paradigm lost?. J. Physiol..

[47] Schmalbruch H., Lewis D.M. (2000). Dynamics of nuclei of muscle fibers and connective tissue cells in normal and denervated rat muscles. Muscle Nerve.

[48] Zhou B., Zheng Y., Li X., Dong H., Yu J., Zou Y., Zhu M., Yu Y., Fang X., Zhou M. (2022). FUS Mutation Causes Disordered Lipid Metabolism in Skeletal Muscle Associated with ALS. Mol. Neurobiol..

[49] Badu-Mensah A., Guo X., McAleer C.W., Rumsey J.W., Hickman J.J. (2020). Functional skeletal muscle model derived from SOD1-mutant ALS patient iPSCs recapitulates hallmarks of disease progression. Sci. Rep..

[50] Steyn F.J., Li R., Kirk S.E., Tefera T.W., Xie T.Y., Tracey T.J., Kelk D., Wimberger E., Garton F.C., Roberts L. (2020). Altered skeletal muscle glucose-fatty acid flux in amyotrophic lateral sclerosis. Brain Commun..

