# Supplementary material for: Increased [18F]DPA-714 Uptake in the Skeletal Muscle of SOD1G93A Mice: A New Potential of Translocator Protein 18 kDa Imaging in Amyotrophic Lateral Sclerosis
Source: Biomolecules. 2025 May 31;15(6):799. doi: 10.3390/biom15060799 (PMC12190601; doi:10.3390/biom15060799)
Supplement: Supplementary file 1 [file biomolecules-15-00799-s001.zip › biomolecules-3640933-supplementary.pdf]

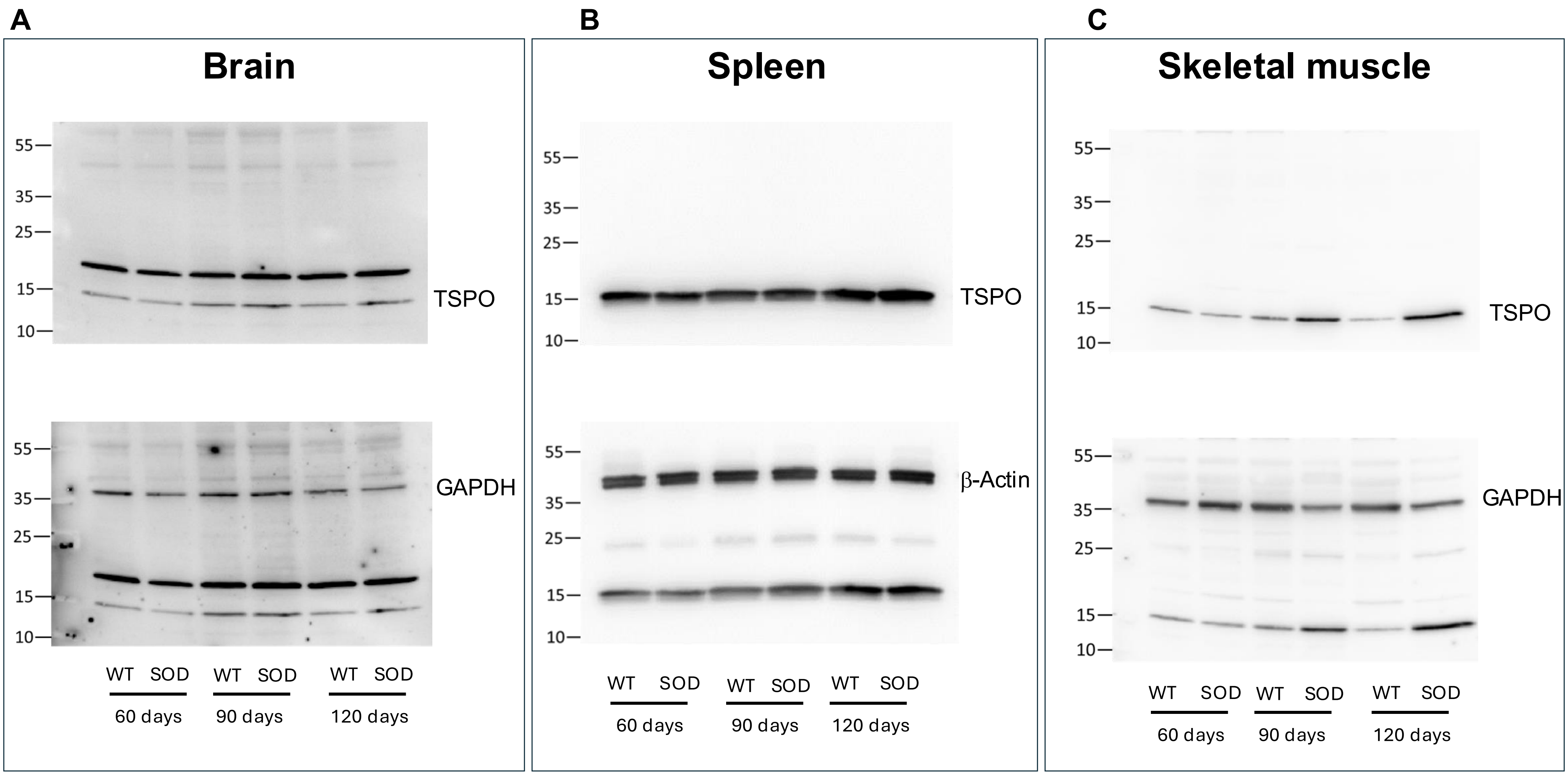

Supplementary Figure. Original Western blot shown in Figure 3. (A) TSPO and GAPDH in brain, (B) TSPO and β-Actin in spleen, and (C) TSPO and GAPDH in skeletal muscle of wild type (WT) and SOD1G93A (SOD) mice at different ages. Membranes were incubated with TSPO (upper panels) and then with GAPDH or β-Actin (lower panels).
